# Tumor mutational burden assessment and standardized bioinformatics approach using custom NGS panels in clinical routine

**DOI:** 10.1186/s12915-024-01839-8

**Published:** 2024-02-20

**Authors:** Célia Dupain, Tom Gutman, Elodie Girard, Choumouss Kamoun, Grégoire Marret, Zahra Castel-Ajgal, Marie-Paule Sablin, Cindy Neuzillet, Edith Borcoman, Ségolène Hescot, Céline Callens, Olfa Trabelsi-Grati, Samia Melaabi, Roseline Vibert, Samantha Antonio, Coralie Franck, Michèle Galut, Isabelle Guillou, Maral Halladjian, Yves Allory, Joanna Cyrta, Julien Romejon, Eleonore Frouin, Dominique Stoppa-Lyonnet, Jennifer Wong, Christophe Le Tourneau, Ivan Bièche, Nicolas Servant, Maud Kamal, Julien Masliah-Planchon

**Affiliations:** 1 Department of Drug Development and Innovation (D3i), Institut Curie, Paris, France; 2 Bioinformatics Core Facility, INSERM U900, Mines Paris Tech, Institut Curie, Paris, France; 3 Department of Medical Oncology, Institut Curie, Paris & Saint Cloud, France; 4 Department of Genetics, Institut Curie, Paris, France; 5 Department of Pathology, Institut Curie, PSL Research University, Paris, France; 6 Department of Pathology, Université Paris-Saclay, UVSQ, Institut Curie, Saint-Cloud, France; 7 Paris-Cité University, Paris, France; 8 INSERM U830, Paris, France; 9 Inserm U900 Research Unit, Saint Cloud, France; 10 Paris-Saclay University, Paris, France; 11 Faculty of Pharmaceutical and Biological Sciences, INSERM U1016, Paris Descartes University, Paris, France

**Keywords:** Tumor mutational burden, Calculation, Immunotherapy, Precision medicine, Molecular Tumor Board

## Abstract

**Background:**

High tumor mutational burden (TMB) was reported to predict the efficacy of immune checkpoint inhibitors (ICIs). Pembrolizumab, an anti-PD-1, received FDA-approval for the treatment of unresectable/metastatic tumors with high TMB as determined by the FoundationOne®CDx test. It remains to be determined how TMB can also be calculated using other tests.

**Results:**

FFPE/frozen tumor samples from various origins were sequenced in the frame of the Institut Curie (IC) Molecular Tumor Board using an in-house next-generation sequencing (NGS) panel. A TMB calculation method was developed at IC (IC algorithm) and compared to the FoundationOne® (FO) algorithm.

Using IC algorithm, an optimal 10% variant allele frequency (VAF) cut-off was established for TMB evaluation on FFPE samples, compared to 5% on frozen samples. The median TMB score for MSS/POLE WT tumors was 8.8 mut/Mb versus 45 mut/Mb for MSI/POLE-mutated tumors. When focusing on MSS/POLE WT tumor samples, the highest median TMB scores were observed in lymphoma, lung, endometrial, and cervical cancers. After biological manual curation of these cases, 21% of them could be reclassified as MSI/POLE tumors and considered as “true TMB high.” Higher TMB values were obtained using FO algorithm on FFPE samples compared to IC algorithm (40 mut/Mb [10–3927] versus 8.2 mut/Mb [2.5–897], *p* < 0.001).

**Conclusions:**

We herein propose a TMB calculation method and a bioinformatics tool that is customizable to different NGS panels and sample types. We were not able to retrieve TMB values from FO algorithm using our own algorithm and NGS panel.

**Supplementary Information:**

The online version contains supplementary material available at 10.1186/s12915-024-01839-8.

## Background

Over the past decade, immunotherapy, and especially immune checkpoint inhibitors (ICIs), has revolutionized the management of several cancer types. Given the durable benefit limited to a minority of patients, the potential toxicities related to ICIs, and the high economic cost of these treatments, predictive biomarkers of response to ICIs are urgently needed.

PD-L1 expression on tumor and/or immune cells using immunohistochemistry has been demonstrated to correlate with ICI efficacy in different cancer types [1–5]. However, PD-L1 expression as a predictive biomarker of efficacy has several limitations, including the lack of sensitivity and specificity, the poor uniformity in the PD-L1 antibody clones, the different scoring methods, and positivity cut-off used [6–9].

Microsatellite instability (MSI) is caused by defects in the mismatch repair genes (therefore also called dMMR and as opposed to microsatellite stable MSS = proficient pMMR) *MSH2*, *MLH1*, *MSH6*, or *PMS2*, leading to an increased rate of mismatch errors [10–12]. Pan-cancer studies have demonstrated the predictive value of MSI (dMMR) on the response to ICIs [13, 14]. However, only 40% of patients with MSI (dMMR) tumors experience an objective response to ICIs. MSI (dMMR) tumors remain rare outside of colorectal and endometrial cancers [15, 16].

*POLE* pathogenic mutations result in ultramutated genomes and were shown to predict response to ICIs [13, 14, 17]. Specifically, mutations in the *POLE* proofreading domain were shown to induce a high tumor mutational burden (TMB). *POLE* mutations remain extremely rare.

TMB is defined as the total number of nucleotidic variants acquired in a tumor and expressed as a number of variants per megabase (Mb). The predictive value of TMB on ICIs efficacy was retrospectively evaluated in the KEYNOTE-158 phase II basket trial of pembrolizumab [18]. High overall response rate was reported in patients with TMB-high tumors defined as ≥ 10 mutations per Mb using the FoundationOne®CDx assay, leading to FDA-approval of pembrolizumab across cancer types in TMB-high tumors. Besides the number of variants/Mb, the type of variants taken into account when estimating the TMB is crucial, because all mutations might not necessarily induce the release of immunogenic peptides and should reflect as close as possible the overall neoantigen load [19]. So far, no consensus exists on TMB calculation method. Besides variations in bioinformatics processing, including variant calling methods and variants filtering, many other factors could influence the TMB estimation [20, 21]. These variations limit the harmonization of TMB calculation and robust effective cut-offs [22–24].

In this study, we aimed to estimate the TMB values from next generation sequencing (NGS) data generated from both FFPE and frozen samples using our own panel and bioinformatics algorithm and to compare the values using the FoundationOne® (FO) algorithm [25, 26]. We eventually propose customizable bioinformatics tool that allows estimating TMB values using other assays than the FO one.

## Results

### Patient characteristics

**Fig. 1:**
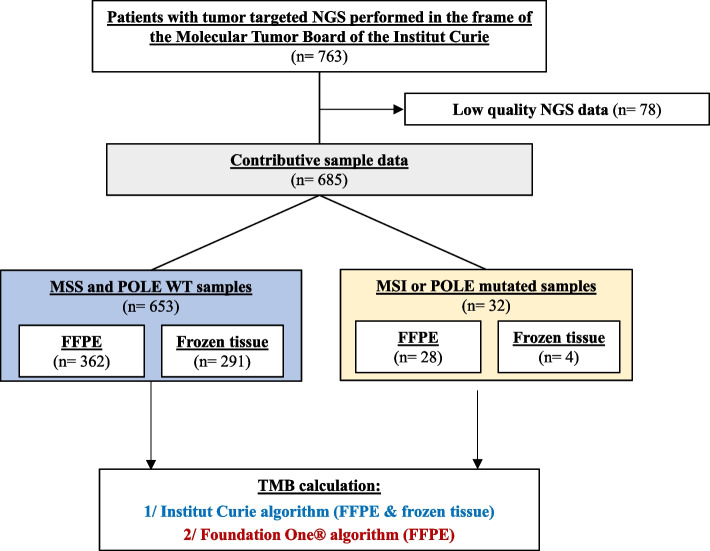
Analysis workflow. MSS, microsatellite stable; WT, wild-type

Tumor samples from 763 patients with various cancer types sequenced through the IC Molecular Tumor Board of using an in-house NGS panel were analyzed in this study. After removing the samples that did not fit the quality criteria (*n* = 78), 685 samples including 390 FFPE and 295 frozen samples from 43 different cancer types were assessed for estimation of the TMB (Table 1 and Fig. 1). In total, 28 samples were MSI high (dMMR) and four samples had a *POLE* mutation (Table 1).

**Table 1:** Cohort characteristics

Cancer type	*n*	Sample type	*n*	MSI/MSS status	*n*	POLE status	*n*
**Breast**	126	FFPE	60	MSI	3	POLE mut	1
		Frozen	66	MSS	123	POLE WT	125
**Colorectal**	72	FFPE	52	MSI	14	POLE mut	1
		Frozen	20	MSS	58	POLE WT	71
**Sarcoma**	72	FFPE	40	MSS	72	POLE WT	72
		Frozen	32				
**Ovarian**	59	FFPE	41	MSI	3	POLE mut	1
		Frozen	18	MSS	56	POLE WT	58
**CNS tumor**	58	FFPE	19	MSS	58	POLE WT	58
		Frozen	39				
**Pancreatic carcinoma**	42	FFPE	35	MSI	2	POLE WT	42
		Frozen	7	MSS	40		
**Endometrial**	28	FFPE	17	MSI	4	POLE WT	28
		Frozen	11	MSS	24		
**Thyroid**	20	FFPE	18	MSS	20	POLE WT	20
		Frozen	2				
**HNSCC**	19	FFPE	15	MSS	19	POLE WT	19
		Frozen	4				
**Lymphoma**	18	Frozen	18	MSS	18	POLE WT	18
**Cholangiocarcinoma**	17	FFPE	12	MSS	17	POLE WT	17
		Frozen	5				
**Lung**	17	FFPE	8	MSS	17	POLE WT	17
		Frozen	9				
**Cervical**	13	FFPE	10	MSS	13	POLE WT	13
		Frozen	3				
**ACUP**	12	FFPE	5	MSI	1	POLE WT	12
		Frozen	7	MSS	11		
**Rhabdoid tumor**	11	FFPE	2	MSS	11	POLE WT	11
		Frozen	9				
**Anal**	10	FFPE	7	MSS	10	POLE WT	10
		Frozen	3				
**Gastric**	10	FFPE	5	MSS	10	POLE WT	10
		Frozen	5				
**Adenoid cystic carcinoma**	8	FFPE	5	MSS	8	POLE WT	8
		Frozen	3				
**Bladder**	7	FFPE	2	MSS	7	POLE WT	7
		Frozen	5				
**Mesothelioma**	7	FFPE	3	MSS	7	POLE WT	7
		Frozen	4				
**Vaginal**	6	FFPE	3	MSS	6	POLE WT	6
		Frozen	3				
**Prostate**	5	FFPE	3	MSS	5	POLE WT	5
		Frozen	2				
**Uveal melanoma**	5	Frozen	5	MSS	5	POLE WT	5
**Cutaneous melanoma**	4	FFPE	2	MSS	4	POLE WT	4
		Frozen	2				
**Sex chord tumor**	4	FFPE	3	MSS	4	POLE WT	4
		Frozen	1				
**Appendix**	3	FFPE	3	MSS	3	POLE WT	3
**Esophageal**	3	FFPE	1	MSS	3	POLE WT	3
		Frozen	2				
**Salivary gland tumor**	3	FFPE	3	MSS	3	POLE WT	3
**UCNT**	3	FFPE	3	MSS	3	POLE WT	3
**GIST**	2	FFPE	2	MSS	2	POLE WT	2
**Neuroendocrine**	2	FFPE	1	MSS	2	POLE WT	2
		Frozen	1				
**Renal**	2	Frozen	2	MSS	2	POLE WT	2
**Vulva**	2	FFPE	2	MSS	2	POLE mut	1
						POLE WT	1
**Craniopharyngioma**	1	FFPE	1	MSS	1	POLE WT	1
**Cutaneous SCC**	1	FFPE	1	MSS	1	POLE WT	1
**Duodenal carcinoma**	1	FFPE	1	MSS	1	POLE WT	1
**Hepatoblastoma**	1	FFPE	1	MSS	1	POLE WT	1
**Leiomyosarcoma**	1	Frozen	1	MSS	1	POLE WT	1
**Peritoneum**	1	FFPE	1	MSS	1	POLE WT	1
**Small bowel carcinoma**	1	FFPE	1	MSI	1	POLE WT	1
**Thymoma**	1	FFPE	1	MSS	1	POLE WT	1
**Waldenstrom**	1	Frozen	1	MSS	1	POLE WT	1
**Other**	6	FFPE	4	MSS	6	POLE WT	6
		Frozen	2				

*FFPE* Formalin-fixed paraffin-embedded, *mut* mutated, *WT* Wild-type, *SCC* Squamous cell carcinoma, *CNS* Central nervous system, *HNSCC* Head and neck squamous cell carcinoma, *ACUP* Adenocarcinoma of unknown primary, *UCNT* Undifferentiated carcinoma of nasopharyngeal type, *GIST* Gastrointestinal stromal tumor

### Development of the in-house TMB estimation algorithm (IC algorithm)

**Fig. 2:**
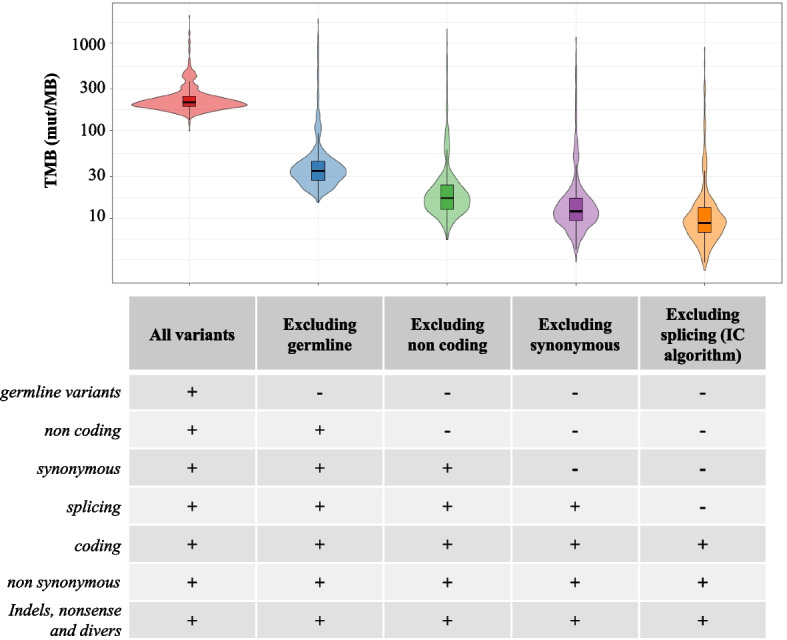
Distribution of TMB score variation among the cohort according to variant filters applied. IC, Institut Curie; Mut, Mutations; TMB, Tumor Mutational Burden

**Fig. 3:**
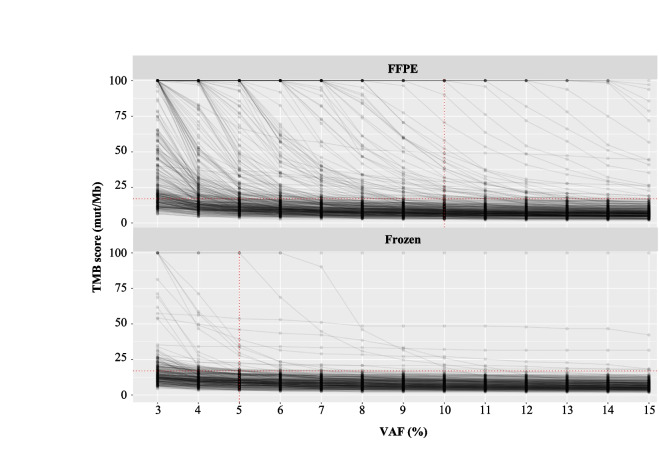
TMB score variation according to variant allele frequency (VAF) cut-off, and sample type (FFPE or frozen). FFPE, formalin-fixed paraffin-embedded; Mut, mutations; TMB, tumor mutational burden; VAF, variant allele frequency

In order to select only potential immunogenic somatic variants, we only considered high-quality, coding, non-synonymous, nonsense, driver variants, and small insertion/deletions (indels), absent from the known polymorphisms/germline database (Fig. 2 and the “ Methods” section). For the same reason, we also decided to determine the minimum VAF to take into account to avoid false positives. To study this parameter, we assessed the evolution of all TMB scores based on the VAF and the sample type (FFPE or frozen), among the MSS/POLE WT cases (Fig. 3). The TMB score inversely correlated with the minimum VAF (Fig. 3 and Additional file 1: Table S1). Higher TMB high scores were observed in FFPE samples compared to frozen samples. TMB scores in frozen tumors rapidly decreased, reaching a plateau for a minimal VAF value around 5%, whereas much heterogeneous results were observed in FFPE tumors with a decrease of TMB scores in much higher VAF cut-offs (Fig. 3). With a minimal VAF threshold fixed at 5%, only 114/362 (31%) FFPE samples had a TMB score between 0 and 10 mut/Mb compared to 147/291 (50%) for frozen samples. Similarly, 44/362 (12%) FFPE samples had a TMB score greater than 100 mut/Mb compared to only 3/291 (1%) for frozen samples (Additional file 1: Table S1).

With a VAF threshold fixed at 10%, 236/362 (65%) FFPE samples had a TMB score ranging from 0 to 10 mut/Mb, compared to 209/291 (72%) for frozen samples. A total of 11/362 (3%) of FFPE samples had a TMB score greater than 100 mut/Mb compared to 1/291 (0.3%) for frozen samples. When moving the VAF threshold from 5 to 10%, 55 FFPE samples switched from a TMB score higher than 30 mut/Mb to lower than 30 compared to only 6 frozen samples (Additional file 1: Table S1).

We then focused on the tumors for which both frozen and FFPE pairs were analyzed (Additional file 2: Fig. S1). For frozen samples, a plateau (which likely represents the true TMB) was reached for a VAF at 5%. For FFPE samples, we were able to distinguish high-quality DNA and low-quality DNA based on pre-analytical parameters as defined in the “ Methods” section. For high-quality FFPE, the steady state was reached with VAF below or around 10%. For low-quality FFPE, the steady state was either reached with a higher VAF or never reached.

We therefore established the minimum VAF threshold used to consider a variant in the TMB estimation to be 5% for frozen samples and 10% for FFPE samples.

### Repartition of TMB scores using IC algorithm

**Fig. 4:**
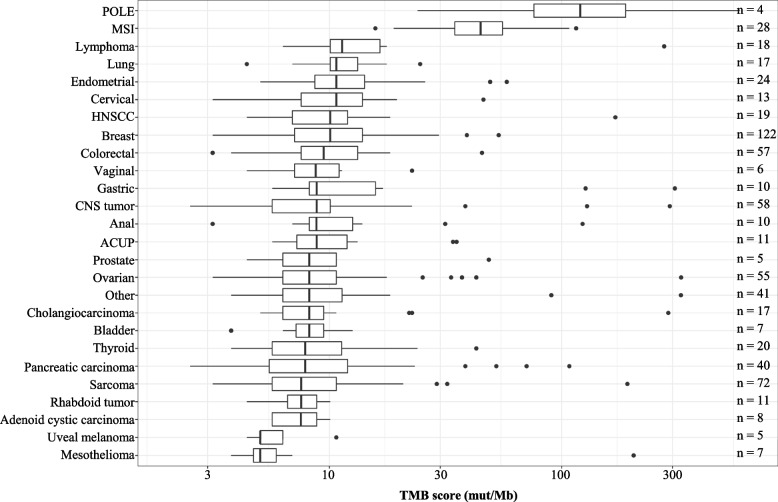
Repartition of TMB scores according to tumor types using the algorithm of the Institut Curie (IC). Tumor types with less than *n* = 5 samples were groups into “Others” in this plot which comprise the following tumor types: cutaneous melanoma, sex chord tumor, appendix, esophageal, salivary gland tumor, UCNT, GIST, neuroendocrine, renal, vulva, craniopharyngioma, cutaneous SCC, duodenal carcinoma, hepatoblastoma, leiomyosarcoma, peritoneum, small bowel carcinoma, thymoma, and Waldenstrom. HNSCC, head and neck squamous cell carcinoma; CNS, central nervous system; ACUP, adenocarcinoma of unknown primary; TMB, tumor mutational burden

We then evaluated the TMB on the 685 contributive samples. The median TMB score calculated with IC algorithm of MSS/POLE-WT tumors was 8.8 mut/Mb [2.5–897] versus 45 mut/Mb [16–584] for MSI/POLE-mutated tumors (Fig. 4 and Additional file 1: Table S2). When focusing on MSS/POLE-WT tumors (*n* = 653), main cancer types analyzed included breast (19%), sarcoma (11%), central nervous system (CNS) (9%), colorectal (9%), and ovarian (8%) cancers. The highest median TMB scores among the MSS/POLE-WT tumors were found in lymphoma (11 mut/Mb [6.3–276]), lung (11 mut/Mb [4.4–24]), endometrial (11 mut/Mb [5.0–58]), and cervical cancer (11 mut/Mb [3.2–46]). The lowest scores among the MSS/POLE-WT tumors were found in uveal melanoma (5.0 mut/Mb [4.4–11]) and mesothelioma (5.0 mut/Mb [3.8–204]) (Fig. 4 and Additional file 1: Table S2).

### Biological curation of TMB-high cases

In order to distinguish true positive TMB-high cases from false positives and to investigate if some cases could be reclassified as MSI-high tumors (dMMR), we focused on the top 10% samples (*n* = 65) with the highest TMB scores among the non-MSI pMMR cases (the MSS/POLE-WT tumors). We removed 8 out of these 65 cases with a bad quality of sequence and considered them as non-contributive for TMB evaluation, leaving 57 TMB high cases. On those cases, 12/57 cases (21%) were found to have either a MSI score ≥ 10% using MSIsensor, a pathogenic variant in one of the MMR genes and/or a mutational signature suggesting a MSI profile, or POLE proofreading deficiency, or APOBEC mutational signature (Additional file 1: Table S3 and Table S4). These samples could be reclassified as MSI/POLE mutated tumors and considered as “true TMB high” cases with a high confidence. For the remaining 45 cases, the high TMB score could not be explained by an MSI status, POLE mutation, or APOBEC signature. For information, we also verified the presence of pathogenic variants (with an allelic ratio ≥ 10%) among 3 candidate genes implicated in DNA damage repair (i.e., *TP53*, *PTEN*, and *ARID1A*). Interestingly, 17/57 cases harbored at least one pathogenic variant in these 3 candidate genes, leaving 28/57 cases (49%) with no explanation for high TMB status.

### TMB scores evaluation using FO algorithm

The TMB score using the FO algorithm was calculated on the 685 contributive samples of the cohort (Additional file 1: Table S2), with a focus on FFPE samples (*n* = 390) to better reproduce the FoundationOne®CDx test conditions. We observed that all TMB values exceeded 10 mut/Mb, the FDA-approved cut-off to consider a tumor TMB-high (Additional file 1: Table S2). When comparing the distribution of TMB scores obtained with the IC algorithm to the one obtained with FO algorithm on the same NGS data derived from all FFPE MSS/POLE-WT tumors (*n* = 362), the median TMB values obtained with IC algorithm were significantly lower compared to the one obtained with the FO algorithm (8.2 mut/Mb [2.5–897] versus 40 mut/Mb [10–3928], *p* < 0.001) (Additional file 2: Fig. S2). Individually, all samples but one had higher TMB from FO algorithm compared to IC algorithm (Additional file 1: Table S2).

## Discussion

We demonstrate that both sample types (FFPE and frozen) and DNA quality (measured with Cp) had an impact on the TMB scores. False positive deamination artifacts (C > T transitions) created by formalin fixation in low-quality FFPE DNA is a well-known effect that can lead to an overestimation of the TMB [20, 24, 27, 28]. This prevents using the same minimum VAF threshold for both FFPE and frozen samples.

Deduplication was not used in our study. Although it could have an impact on the variant calling accuracy, and thus affect the TMB score [20, 29], other studies showed that deduplication was not always mandatory [30, 31] or could be overcome by applying a 10% VAF threshold [20, 32]. We have demonstrated that the use of UMI-based deduplication did not impact our results by calculating the VAFs of all variants with or without UMI processing and computing the correlation between VAFs values for each patient. An average correlation of 0.952 for the FFPE samples and 0.983 for the frozen samples demonstrated that the UMI processing has very little impact on the VAFs (Additional file 2: Fig. S3). This is in line with other publications [30, 31, 33].

Based on our analysis of more than 750 samples and previous recommendations [20, 34], we proposed a 10% VAF cut-off for FFPE samples and a 5% cut-off for frozen samples. The high TMB scores found in FFPE samples, possibly due to fixation artifacts, represents a clinical reality to be dealt with for routine TMB calculation, across all laboratories [20, 24, 27, 28]. In this study, we propose a general algorithm with appropriate filters and threshold to limit the impact of such artifacts, but a manual curation step for this kind of samples will always be unavoidable. Using a fixed threshold allows to (i) simplify the variant calling process, making it more standardized and easier to implement across different samples and studies, (ii) provide consistency when comparing TMB across samples, and (iii) homogenize the interpretation of results. These points are particularly important in clinical settings where uniformity in methodology is required.

To overcome this problem upstream of the analysis, we applied the most rigorous possible filters to remove the false positives while preserving the true variants. Other possibilities might include the implementation of dedicated computational algorithm to rectify formalin-induced artifacts for FFPE samples [35] or optimization of the chemistry with the use of enzymes involved in base excision repair before library preparation [36].

Using the FO algorithm, all TMB scores exceeded 10 mut/Mb, which differs from what has been reported in the literature [25, 37]. These results suggest that the level of information provided by FoundationOne® does not enable to reproduce their algorithm and consequently to directly transpose the FO algorithm to other targeted NGS panels.

The choice of variants to take into account when estimating the TMB is crucial, because all mutations do not necessarily induce the release of immunogenic peptides, and should reflect as close as possible the overall neoantigen load [19]. As targeted panels include mainly cancer genes, which are more likely to be mutated in the tumor, some methods have been proposed to filter out known cancer variants for TMB quantification. We chose to keep cancer hotspots variants in our algorithm for the TMB estimation, since they could also generate immunogenic peptides. We also chose to filter out synonym and non-coding variants as they are unlikely to generate neoepitopes and the size of the coding sequence of our in-house NGS panel is sufficient to assure TMB reliability [26]. Compared to whole exome sequencing, NGS panels are not constantly associated with the germline paired DNA sequencing. This requires a substantial methodology to filter out the polymorphisms that come from the germline and hence might not induce an immune response. Germline variants are commonly filtered using databases of known germline mutations. Some algorithms use complementary germline removal algorithm such as somatic-germline-zygosity [38]. Here, due to partially available information on the SGZ algorithm proposed by FoundationOne® as part of their commercial product (FoundationOne®CDx), we used different databases of known germline mutations as references (Exac, 1000G or GnomaD all ethnicities) to remove as many germline variants as possible and only retain private or extremely rare germline polymorphisms, which may increase TMB score [39].

Overall, several parameters including biological factors to pre-analytics, sequencing, and bioinformatics can impact the TMB scores estimation, explaining the diversity of published TMB algorithms, the heterogeneity of the results, and the complexity to harmonize methods [20]. The bioinformatics tool used in this study is freely available for the community and highly customizable to fit different targeted NGS panels and sample types (both FFPE and frozen). Other tools for TMB calculation have been developed and reported in the literature. Their applicability still needs to be tested, since they often require to have paired targeted NGS and WES data for each patient. In addition, the sample type (frozen or FFPE) and quality are not taken into account in the estimation [33, 40].

The TMB estimation using our algorithm revealed variations in the medians and ranges across tumor types, with the highest median TMB score found in MSI/POLE-mutated tumors. Our results are in line with previous reports in the literature [18, 25, 37, 41]. We observed that some tumors harbored very high TMB scores, although not associated with MSI status (dMMR) or *POLE* mutations at first glance. After biological manual curation of these cases, 21% of them could be reclassified as MSI/POLE tumors and considered as “true TMB high” with a high level of confidence, and 30% had at least one pathogenic variant among 3 candidate genes implicated in DNA damage repair that could be related to high TMB (i.e., *TP53*, *PTEN*, and *ARID1A*) [42–44]. However, for the remaining cases, the high TMB scores could not find a biological explanation. The more detailed manual observation of TMB-high cases represents the reality of TMB status validations carried out by the experts within the framework of clinical routine use.

## Conclusions

In conclusion, we show that the TMB values obtained from the same NGS data but with different calculation methods are not comparable. In order to optimize the implementation of TMB as a robust predictive biomarker of efficacy of ICIs, the determination of the method to be used to identify the right threshold is key. Studies from cohorts of patients treated with ICIs will be needed to identify these thresholds as well as studies on larger series of matched FFPE and Frozen samples to determine the most optimal way to avoid artifacts in the calculation of TMB (i.e., using different algorithms with a possible different VAF cut-off for variant calling, or using different cut-offs on TMB values for high or low statuses according to a FFPE or frozen sample).

## Methods

### Patient selection

Patients with recurrent and/or metastatic cancers whose tumor was sequenced in the frame of Molecular Tumor Board of the Institut Curie (IC) [45] were included in this study. Informed consent with regard to the collection of tumor samples and molecular analysis was obtained from patients within the IC institutional general consent signed by every patient treated at the IC.

### In-house next generation sequencing panel

Samples were sequenced using an in-house NGS panel covering 1.6 Mb. Indexed paired-end libraries of tumor DNA were performed using the Agilent Sureselect XT-HS library prep kit. Fifty nanograms of input DNA were used to build the libraries according to manufacturer’s protocol. Libraries were sequenced on the NovaSeq 6000 (Illumina) Sp 2 × 100 bp flow cell.

### Bioinformatics

After tumor DNA sequencing, bioinformatics analyses were performed as detailed below in order to detect single-nucleotide variants (SNVs) and indels, microsatellite instability statuses, mutational signatures, and TMB scores (detailed in Additional file 3: Supplementary Methods and above).

### Variant calling

Variant calling of both SNVs and indels was carried out on the aligned sequencing data as previously described [46]. Annotations from several databases [RefSeq [47], dbsnp v150 [48], COSMIC v86 [49], 1000 g project 08/2015 version [50], ESP6500 [51] gnomAD (all and ethnicities) [39], ICGC v21 [52], and dbnsfp v35 [53] predictions] were provided by Annovar (04/16/2018 version, Wang *et al.* [54]).

### TMB calculation

After removing low NGS quality samples, i.e., samples with < 20 million sequencing reads or < 15% of the captured regions sequenced above 1000X, the TMB values were calculated using two different algorithms: (1) the FO algorithm on FFPE samples and (2) our IC algorithm on all samples including both FFPE and frozen (Fig. 1).

FoundationOne® (FO) TMB algorithm was reproduced based on the Summary of Safety and Effectiveness (https://www.accessdata.fda.gov/cdrh_docs/pdf17/P170019S016B.pdf). Low-quality variants were removed based on the absence of “PASS” tag from varScan2 variant calling results. Germline variants were also removed from the vcf files using the somatic-germline-zygosity (SGZ) algorithm (v1.0.0) [38] as well as polymorphisms database (variants found in 1000 Genomes or Exac [55] databases for all ethnicities with a minor allelic frequency (MAF) higher than 0.1%). Non-coding variants and driver mutations found at least once in COSMIC database were also removed. Hence, all coding variants including synonymous, splicing (defined as every intronic nucleotide within 2 bp at the exon/intron boundaries), and indels were considered for the final TMB calculation if their VAF was higher than 5% and the depth of coverage higher than 100X. Of note, with the information provided by FoundationOne®, we were not able to reproduce their exact capture regions and thus based our TMB calculation on our own design and dividing the number of variants by 1.6 Mb to obtain the number of mutations per Mb.

For IC TMB algorithm, recurrent variants detected in more than 15% of the samples within the same sequencing run were considered as false positive and removed from the TMB calculation. Polymorphisms found in 1000 Genomes, Gnomad, or Exac databases for all ethnicities with a MAF higher than 0.1% were also removed. Given that the goal of TMB is to identify likely immunogenic tumors that ultimately could respond to ICI, and that only somatic, acquired, coding variants encode potential neoantigens, we decided to consider in the IC algorithm the coding, non-synonymous, and indels variants but to remove non-coding, synonymous, and splice (defined as every intronic nucleotide within 2 bp at the exon/intron boundaries) variants. Finally, only variants with a VAF higher than 5% for frozen samples or 10% for FFPE samples and a depth of coverage higher than 100X were considered for TMB estimation.

In order to standardize the TMB estimation, we developed a bioinformatics tool named pyTMB that can be applied to any sequencing data type. pyTMB can be easily installed with conda either directly from the source code (https://github.com/bioinfo-pf-curie/TMB) or from the bioconda channel. PyTMB v1.1.0 has been used by this study (10.5281/zenodo.10573735). pyTMB requires a list of annotated variants and successively applies the different filters that can then be adapted by the users. The version 1.1.0 supports.vcf files generated with the Mutect2 and Varscan2 tools and annotated with either ANNOVAR or snpEff (Table 2 and Additional file 3: Supplementary Methods).

**Table 2:** Filters applied for TMB calculation with Foundation One ® (FO) algorithm and Institut Curie (IC) algorithm

	FoundationOne® algorithm	Institut Curie algorithm
Metrics		
Low-quality variant removal	YES PASS tag from VarScan2	YES Intra-run recurrence
Minimum VAF	5%	5% for frozen tumors 10% for FFPE tumors
Minimum depth of coverage	100X	100X
Germline removal	SGZ algorithm And SNP from 1000 Genomes or Exac with a MAF > 0.1% were filtered	SNP from 1000 Genomes, Gnomad or Exac with a MAF > 0.1% were filtered
Variants types		
Drivers	Excluded COSMIC	Included
Synonymous	Included	Excluded
Splicing	Included	Excluded
Indels	Included	Included
Nonsense	Included	Included
Non-coding	Excluded	Excluded

The FO algorithm was applied on FFPE samples only as required by the FoundationOne®CDx test, while the IC algorithm was used on both frozen and FFPE samples

*FFPE* Formalin-fixed paraffin-embedded, *VAF* Variant allele frequency, *SGZ* Somatic-germline-zygosity, *COSMIC* Catalogue of Somatic Mutations in Cancer, *MAF* Mutation allele frequency, *SNP* Single-nucleotide polymorphism

### Biological curation of TMB high cases

To avoid false positives related to bad quality DNA, we focused on the top 10% samples with the highest TMB scores (corresponding to a TMB > 17.5 mut/Mb using the IC algorithm) among the non-MSI (pMMR) cases (MSS/POLE WT tumors). To further investigate the high TMB cases, we individually assessed: (i) the MSI score using MSI sensor, (ii) mutations in MMR-related genes (e.g., in *MSH2*, *MSH1*, *MSH6*, or *PMS2* gene), and (iii) the presence of MMR or APOBEC-related mutational signatures (see Additional file 3: Supplementary Methods).

## Notes

### Role of the funder

The authors are all part of the Institut Curie which provided the resources for the personnel as well as the equipment, reagents, materials, and structures needed for the Molecular Tumor Board and for the analyses. Amgen France, La Ligue Contre le Cancer, and Cancéropole Ile-de-France provided funding for reagents, sample processing, and personnel resources through grants.

### Supplementary Information

**Additional file 1: Table S1.** Detailed TMB score variation according to variant allele frequency (VAF) cut-off and to sample type (FFPE or frozen). **Table S2.** Detailed TMB evaluation across the 685 contributive tumor samples. FFPE = Formalin-Fixed Paraffin-Embedded; mut = mutated; WT = Wild-Type; MSS = MicroSatellite Stable; MSI = MicroSatellite Instable; SCC = Squamous Cell Carcinoma; CNS = Central Nervous System; HNSCC = Head and Neck Squamous Cell Carcinoma; ACUP = AdenoCarcinoma of Unknown Primary; UCNT = Undifferentiated Carcinoma of Nasopharyngeal Type; GIST = Gastrointestinal Stromal Tumor. **Table S3.** Focus on TMB high cases including the evaluation of MSI score, MMR-related gene mutations, and MMR-related mutational signatures. * : pathogenic variants (with an allelic ratio ≥10%) among 3 candidate genes implicated in DNA damage repair (i.e., TP53, PTEN, and ARID1A). MSS = MicroSatellite Stable; MMR = MisMatch Repair. **Table S4.** MMR genes mutational variants detected in 3 samples with high TMB. MMR = MisMatch Repair; MSI = MicroSatellite Instable.

**Additional file 2: Fig. S1.** TMB score variation according to DNA sample quality and according to sample type (FFPE or frozen) in 10 sample pairs. FFPE = Formalin-Fixed Paraffin-Embedded; TMB = Tumor Mutational Burden; VAF = Variant Allele Frequency. **Fig. S2.** TMB scores according to the algorithm of the Institut Curie (IC) and FoundationOne® (FO), obtained from the same NGS data of 362 MSS/POLE WT FFPE pan-cancer samples. *** *p* < 0.001 using Wilcoxon signed-rank test. FFPE = Formalin-Fixed Paraffin-Embedded; MSS = MicroSatellite Stable. **Fig. S3.** Computational analysis of VAFs correlation with or without UMI processing in FFPE and frozen samples for each patient.

**Additional file 3: Supplementary Methods.** TMB calculation method and parameters applied for each algorithm.

**Additional file 4.**

## Data Availability

All data generated or analyzed during this study are included in this published article, its supplementary information files, and publicly available repositories. NGS and clinical data were obtained from tumor samples from 763 patients treated at Institut Curie. Supporting data values for *n* < 6 individual data values reported in the figures are detailed in the Supporting data values file. Source code for pyTMB can be found on GitHub (https://github.com/bioinfo-pf-curie/TMB) and Zenodo (10.5281/zenodo.10573735).

## Abbreviations

ACUP: Adenocarcinoma of unknown primary
COSMIC: Catalogue of Somatic Mutations in Cancer
CNS: Central nervous system
FDA: Food and Drug Administration
FFPE: Formalin-fixed paraffin-embedded
FO: FoundationOne
GIST: Gastrointestinal stromal tumor
HNSCC: Head and neck squamous cell carcinoma
IC: Institut Curie
ICIs: Immune checkpoint inhibitors
Indels: Small insertion/deletions
MAF: Mutation allele frequency
MMR: Mismatch repair
MSI: Microsatellite instability
MSS: Microsatellite stable
Mut: Mutated/mutations
NGS: Next generation sequencing
SCC: Squamous cell carcinoma
SGZ: Somatic-germline-zygosity
SNP: Single-nucleotide polymorphism
TMB: Tumor mutational burden
UCNT: Undifferentiated carcinoma of nasopharyngeal type
VAF: Variant allele frequency
WT: Wild-Type
