# Supplementary figures and images for: Tumor mutational burden assessment and standardized bioinformatics approach using custom NGS panels in clinical routine
Source: BMC Biol. 2024 Feb 20;22:43. doi: 10.1186/s12915-024-01839-8 (PMC10880437; doi:10.1186/s12915-024-01839-8)

## Slide 1
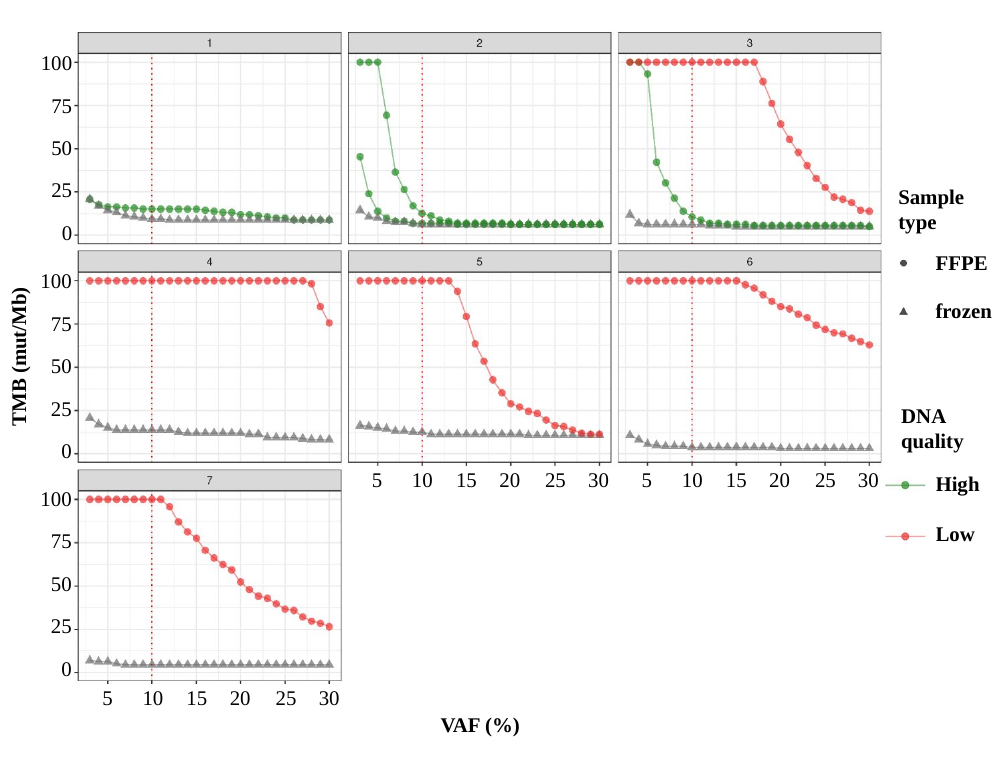

Sample type
TMB (mut/Mb)
100
75
50
25
0
FFPE
100
75
50
25
0
frozen
DNA quality
5
10
15
20
25
30
5
10
15
20
25
30
High
100
75
50
25
0
Low
5
10
15
20
25
30
VAF (%)

Supplement: Supplementary file 2 — Additional file 2: Fig. S1. TMB score variation according to DNA sample quality and according to sample type (FFPE or frozen) in 10 sample pairs. FFPE = Formalin-Fixed Paraffin-Embedded; TMB = Tumor Mutational Burden; VAF = Variant Allele Frequency. Fig. S2. TMB scores according to the algorithm of the Institut Curie (IC) and FoundationOne® (FO), obtained from the same NGS data of 362 MSS/POLE WT FFPE pan-cancer samples. *** p < 0.001 using Wilcoxon signed-rank test. FFPE = Formalin-Fixed Paraffin-Embedded; MSS = MicroSatellite Stable. Fig. S3. Computational analysis of VAFs correlation with or without UMI processing in FFPE and frozen samples for each patient. [file 12915_2024_1839_MOESM2_ESM.zip › Figure S1_12052023.pptx]

## Slide 1
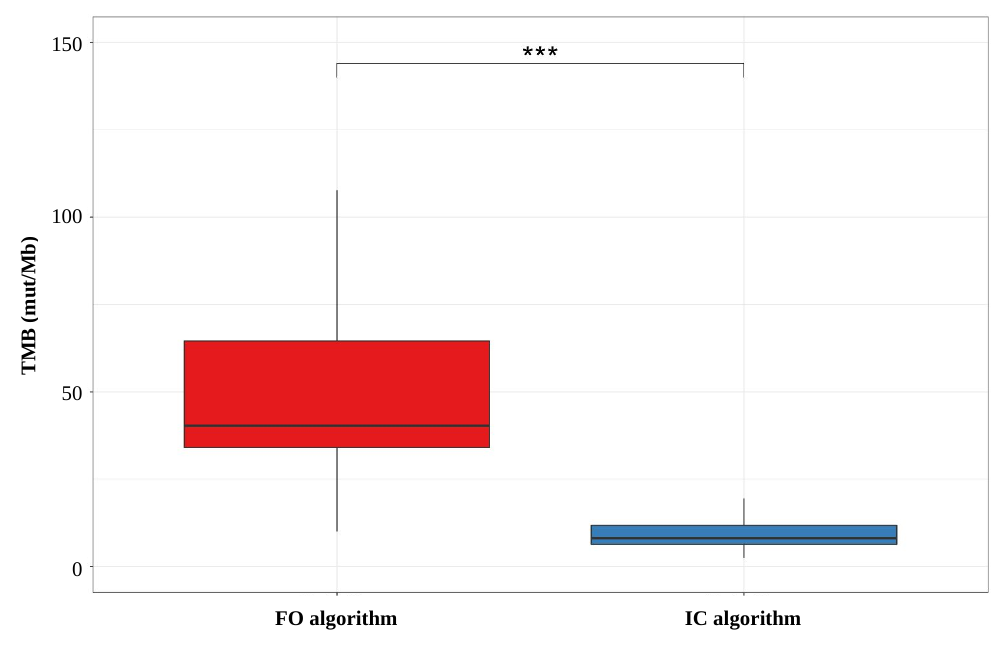

150
100
50
0
TMB (mut/Mb)
FO algorithm
IC algorithm

Supplement: Supplementary file 2 — Additional file 2: Fig. S1. TMB score variation according to DNA sample quality and according to sample type (FFPE or frozen) in 10 sample pairs. FFPE = Formalin-Fixed Paraffin-Embedded; TMB = Tumor Mutational Burden; VAF = Variant Allele Frequency. Fig. S2. TMB scores according to the algorithm of the Institut Curie (IC) and FoundationOne® (FO), obtained from the same NGS data of 362 MSS/POLE WT FFPE pan-cancer samples. *** p < 0.001 using Wilcoxon signed-rank test. FFPE = Formalin-Fixed Paraffin-Embedded; MSS = MicroSatellite Stable. Fig. S3. Computational analysis of VAFs correlation with or without UMI processing in FFPE and frozen samples for each patient. [file 12915_2024_1839_MOESM2_ESM.zip › Figure S2_12052023.pptx]

## Slide 1
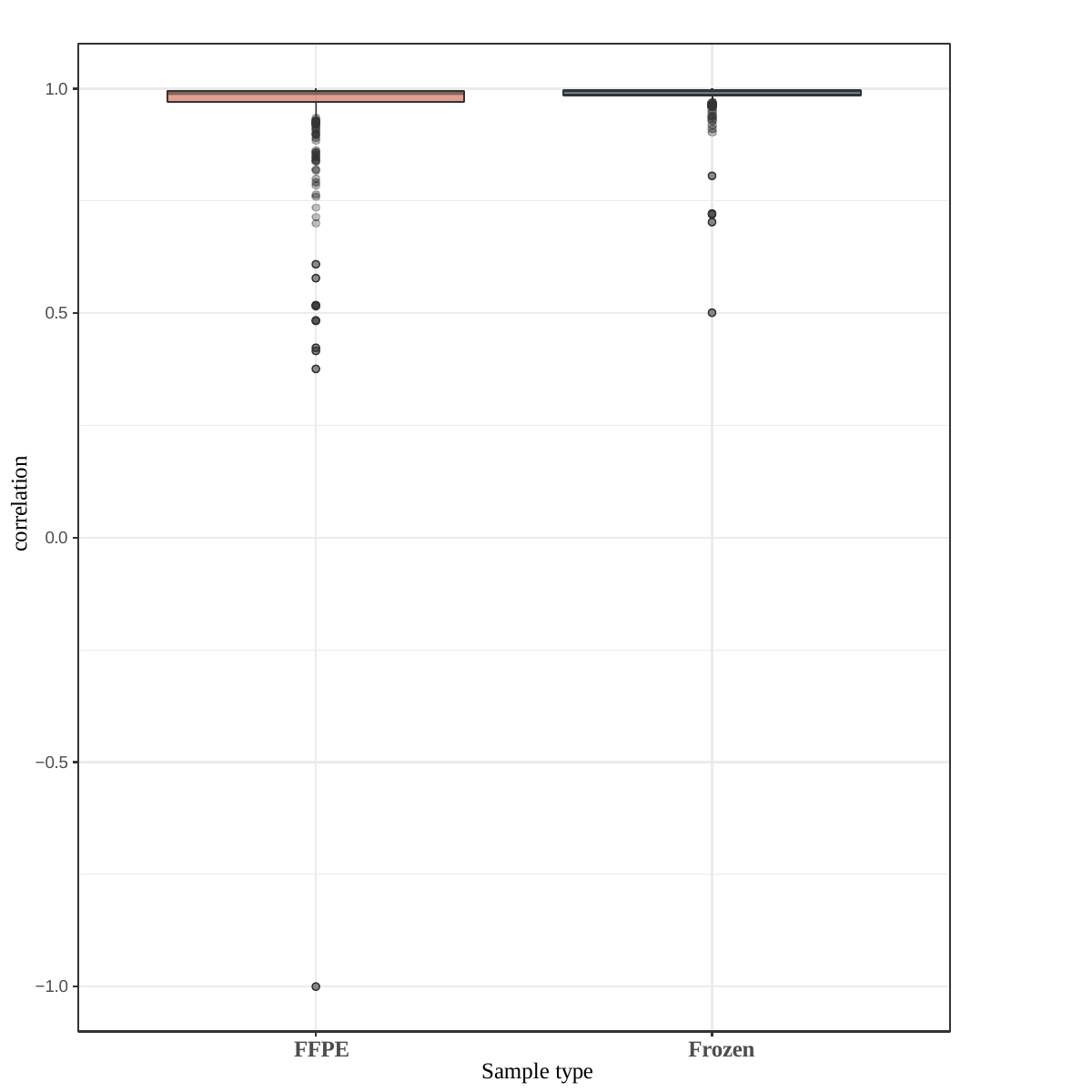

1.0
0.5
correlation
0.0
−0.5
−1.0
FFPE
Frozen
Sample type

Supplement: Supplementary file 2 — Additional file 2: Fig. S1. TMB score variation according to DNA sample quality and according to sample type (FFPE or frozen) in 10 sample pairs. FFPE = Formalin-Fixed Paraffin-Embedded; TMB = Tumor Mutational Burden; VAF = Variant Allele Frequency. Fig. S2. TMB scores according to the algorithm of the Institut Curie (IC) and FoundationOne® (FO), obtained from the same NGS data of 362 MSS/POLE WT FFPE pan-cancer samples. *** p < 0.001 using Wilcoxon signed-rank test. FFPE = Formalin-Fixed Paraffin-Embedded; MSS = MicroSatellite Stable. Fig. S3. Computational analysis of VAFs correlation with or without UMI processing in FFPE and frozen samples for each patient. [file 12915_2024_1839_MOESM2_ESM.zip › Figure S3_12052023.pptx]
