# Supplementary material for: Tumor mutational burden assessment and standardized bioinformatics approach using custom NGS panels in clinical routine
Source: BMC Biol. 2024 Feb 20;22:43. doi: 10.1186/s12915-024-01839-8 (PMC10880437; doi:10.1186/s12915-024-01839-8)
Supplement: Supplementary file 3 — Additional file 3: Supplementary Methods. TMB calculation method and parameters applied for each algorithm. [file 12915_2024_1839_MOESM3_ESM.docx]

**Supplementary materials and methods**

## **Tumor sample collection and processing**

Analyses are performed by giving preference to frozen samples and tumor cellularity was determined from cryosections after Hematoxylin and Eosin (H&E) staining. For FFPE samples, tumor cells content was evaluated on H&E slides and allowed macrodissection of the sample to increase tumor cellularity when possible. For both FFPE and frozen samples, the evaluation of tumor cellularity was assessed by a medical pathologist scoring the percentage of the overall tumor cellularity over the surface of the tissue sample. A minimum of 10% tumor cell fraction was accepted for further molecular analyses.

## **Tumor DNA extraction**

DNA from frozen samples were extracted by the Biological Resource Center of Institut Curie, using Phenol:Chloroform:Isoamyl Alcohol (Invitrogen) under manufacturer’s instructions. For FFPE samples, DNA was extracted from the tissue using the NucleoSpin® 8 Tissue Core kit (Macherey Nagel) following manufacturer’s instructions. FFPE derived samples were qualified by a PCR HRM using primers for *KRAS* exon 2 as previously described. This method allows to get a Crossing point value (Cp) corresponding to the DNA quality. Good quality DNA was defined as Cp ≤ 34 with a concentration > 0.2 ng/µl. Low DNA quality was defined as CP ≥ 34 with a concentration < 0.2 ng/µl or a CP > 35.

## **Bioinformatics**

## ***Reads mapping***

First, reads were mapped using ‘BWAmem’ software (v0.7.15)(Chakravarty et al. 2017) on the human reference genome (hg19 assembly) using default parameters. As a second quality control, statistics regarding the mapping (percentage of aligned reads total and falling into the capture, percentage of PCR duplicates) and the capture coverage were produced using a combination of ‘SAMtoolsflagstat’, ‘BEDtools coverage’ and ‘PicardTools MarkDuplicates’ (« Picard Tools - By Broad Institute » s. d.; Danecek et al. 2021; Quinlan et Hall 2010).

Deduplication was not used to discard PCR duplicates. UMI (Unique Molecular Identifiers) was used in the preparation of the library but not for the bioinformatics processing of the data as we do not find any significant impact of the UMIs on the variant allele frequencies (Additional file 2: Fig. S1).

## ***Microsatellite instability***

MSIsensor2 (<https://github.com/niu-lab/msisensor2commit>, ebdbf42, niu-lab), uses machine learning models to figure out the MSI status for a distribution per microsatellite. Considering a selection of 74 microsatellites pan-cancer selected to be the most unstable (Hause et al. 2016), percentages of unstable *loci* (number of MSI sites / all valid sites) were computed per sample. For all samples, MSI score cut-off value of 15% was used to consider MSI status and for the Biological curation of TMB high cases, the range of value considered was 10-15%. A manual curation was also performed to validate the MSI status of those samples.

## ***Mutational signatures***

Palimpsest (v2.0.0) (Shinde et al. 2018) was used to extract known single base substitution (SBS) mutational signatures (COSMIC database – version 3, release v89) (Alexandrov et al. 2020) from the input tumors. Somatic variants, including synonymous variants, passing the filters (minimum of 10 variants/tumor) were used to extract mutational signatures (using NMF, non-negative matrix factorization).

## ***TMB evaluation***

1. Variant Calling / Annotation

Annotations from several databases (RefSeq, dbsnp v150, COSMIC v86, 1000g project 08/2015 version, ESP6500, gnomAD (all and ethnies), ICGC v21, and dbnsfp v35 predictions) were provided by Annovar (04/16/2018 version, Wang *et al.*¸ 2010) to annotate small variants.

1. Foundation One TMB algorithm

Foundation One TMB calculation was reproduced based on the Summary of Safety and Effectiveness (page9 – <https://www.accessdata.fda.gov/cdrh_docs/pdf17/P170019S016B.pdf>). Germline variants were first removed from the vcf files using the Somatic Germline Zygosity (SGZ) algorithm (v1.0.0, <https://github.com/jsunfmi/SGZ>). Then low quality variants (without “PASS” tag from varscan2) were removed. Polymorphic variants (found in 1000 Genomes or Exac databases) with a Minor Allelic Frequency (MAF) of 0.1% were filtered. Also, non coding variants and known and likely driver mutations found in COSMIC database were removed. Coding, synonymous, non synonymous, splicing and indels variants were considered for the final TMB calculation with the following thresholds:

- Variant allele frequency: 5%

- minimum depth of coverage : 100

- minimum depth for alternative allele : 5

Finally, the number of remaining variants was divided by the size of the capture to obtain the number of mutations per Mb of captured genome.

1. Institut Curie TMB
   1. Algorithm

For Institut Curie TMB, recurrent variants detected within the same run were first considered as false positive and discarded. Low quality, non coding, synonymous, splice and polymorphic (found in 1000 Genomes, Gnomad and Exac databases) with a MAF of 0.1% variants were filtered. Coding, non-synonymous and indels variants were considered. In addition, the following thresholds were applied:

- Variant allele frequency: 5% for frozen samples or 10% for FFPE samples to limit the impact of deamination artifacts on the TMB score.

- minimum depth of coverage : 100

- minimum depth for alternative allele : 5 for frozen and 10 for FFPE samples.

Finally, the number of remaining variants was divided by the size of the capture to obtain the number of mutations per megabase (Mb) of captured genome.

- 1. Implementation

In order to ease the reproducibility and the standardization of TMB calculation, we developed a new bioinformatics tool named pyTMB which aims at calculating a TMB score, following a defined algorithm and a size of panel (<https://github.com/bioinfo-pf-curie/TMB>). It can be easily install using conda and is available in the bioconda channel (https://anaconda.org/bioconda/tmb). This tool has been developed to be as flexible as possible. It starts from a list of annotated variants (.vcf) and will successively applied the different filters following the user defined algorithm. The current version supports vcf files generated with the Mutect2 and Varscan tools and annotated with ANNOVAR or snpEff.

1. Identification of MSI and POLE samples

MSI status was assessed with msi-sensor2 (https://github.com/niu-lab/msisensor2 , commit ebdbf42, niu-lab). Samples with 15% of unstable loci were considered MSI High (MSI-H). A manual curation was also performed to validate those samples.

POL-E mutated samples were identified with in-house bash scripts and manual curation.

1. Filtered samples

Samples with less than 20 million sequencing reads or less than 15% of the captured regions sequenced above 1000X were considered as low quality samples and were removed from the analysis, to ensure that > 95% of the capture is covered at more than 100X.

1. Additionnal informations
   1. list of variants considered :

For each variant category, specific variant annotation based on Annovar were considered (<https://annovar.openbioinformatics.org/en/latest/user-guide/gene/>) .

- coding : exonic

- splicing : splicing and ncRNA_splicing

- non coding : intronic, UTR5, UTR3, ncRNA_exonic, ncRNA_intronic, ncRNA_splicing, upstream, intergenic, downstream,

- synonymous : synonymous_SNV

- Non synonymous : nonsynonymous_SNV

- 1. List of annotation databases used for filtering polymorphic variants
- Cosmic
- 1000 Genomes
- Exac
- Gnomad
  1. Bash command to run pyTMB with Institut Curie algorithm

if [ "$type" = "FFPE" ] || [ "$type" = "unknown" ]; then

echo "type:" $type

python $PYTMB/pyTMB.py \

-i $OUTPUT_DIR/VCF/$ID.hg19_multianno.rec.vcf\

--sample $ID \

--bed $BED \

--vaf 10 --maf 0.001 --minDepth 100 --minAltDepth 5 \

--filterLowQual --filterSplice --filterNonCoding --filterSyn \

--filterRecurrence --filterPolym --polymDb 1k,gnomad,exac \

--dbConfig $PYTMB/config/annovar_dragon.yml \

--varConfig $PYTMB /config/varscan2.yml > $OUTPUT_DIR_CURIE/$ID"."$type"_tmb.txt"

elif [ "$type" = "frozen" ] || [ "$type" = "ctDNA" ]; then

echo "type:" $type

python $PYTMB/pyTMB.py \

-i $OUTPUT_DIR/VCF/$ID.hg19_multianno.rec.vcf \

--sample $ID \

--bed $BED \

--vaf 5 --maf 0.001 --minDepth 100 --minAltDepth 5 \

--filterLowQual --filterSplice --filterNonCoding --filterSyn \

--filterRecurrence --filterPolym --polymDb 1k,gnomad,exac \

--dbConfig $PYTMB/config/annovar_dragon.yml \

--varConfig $PYTMB/tmb/config/varscan2.yml > $OUTPUT_DIR_CURIE/$ID"."$type"_tmb.txt"

else

echo "other:" $type

fi

- 1. Bash command to run pyTMB with Foundation One like algorithm

if [ "$type" = "FFPE" ] || [ "$type" = "unknown" ] || [ "$type" = "frozen" ] || [ "$type" = "ctDNA" ]; then

python $PYTMB/pyTMB.py \

-i $OUTPUT_DIR_F1/$ID"_fmi.sgz.somatic.vcf"\

--bed $BED \

--sample $ID \

--vaf 5 --maf 0.001 --minDepth 100 --minAltDepth 5 \

--filterNonCoding --filterLowQual \

--filterPolym --polymDb exac,1k --filterCancerHotspot \

--dbConfig $PYTMB/tmb/config/annovar_dragon.yml \

--varConfig $PYTMB/tmb/config/varscan2.yml > $OUTPUT_DIR_F1/$ID"_fmi_tmb.txt"

## **Statistical analysis**

Using R software (v3.6.0), a Wilcoxon signed-rank test was performed to assess the difference in the distribution of TMB values between IC and FO algorithms. A Kruskal-Walis non-parametric test was performed to compare TMB values originating from different cancer types. For the focus on TMB high cases and comparison of groups, GRAPHPAD PRISM (GraphPad Software, San Diego, CA, USA) 4 software was used to apply Kruskal–Wallis test followed by Dunn’s test. p < 0.05 was considered as statistically significant.
